# Women’s Participation in Decision-Making in Maternity Care: A Qualitative Exploration of Clients’ Health Literacy Skills and Needs for Support

**DOI:** 10.3390/ijerph18031130

**Published:** 2021-01-27

**Authors:** Laxsini Murugesu, Olga C. Damman, Marloes E. Derksen, Danielle R. M. Timmermans, Ank de Jonge, Ellen M. A. Smets, Mirjam P. Fransen

**Affiliations:** 1 Department of Public and Occupational Health, Amsterdam Public Health, Amsterdam UMC, University of Amsterdam, 1105 AZ Amsterdam, The Netherlands; m.e.derksen@amsterdamumc.nl (M.E.D.); m.p.fransen@amsterdamumc.nl (M.P.F.); 2 Department of Public and Occupational Health, Amsterdam Public Health, Amsterdam UMC, Vrije Universiteit Amsterdam, 1081 HV Amsterdam, The Netherlands; o.damman@amsterdamumc.nl (O.C.D.); drm.timmermans@amsterdamumc.nl (D.R.M.T.); 3 Department of Midwifery Science, AVAG, Amsterdam Public Health, Amsterdam UMC, Vrije Universiteit Amsterdam, 1081 HV Amsterdam, The Netherlands; ank.dejonge@amsterdamumc.nl; 4 Department of Medical Psychology, Amsterdam Public Health, Amsterdam UMC, University of Amsterdam, 1105 AZ Amsterdam, The Netherlands; e.m.smets@amsterdamumc.nl

**Keywords:** decision-making, client participation, maternity care, health literacy, needs assessment

## Abstract

Shared decision-making requires adequate functional health literacy (HL) skills from clients to understand information, as well as interactive and critical HL skills to obtain, appraise and apply information about available options. This study aimed to explore women’s HL skills and needs for support regarding shared decision-making in maternity care. In-depth interviews were held among women in Dutch maternity care who scored low (*n* = 10) and high (*n* = 13) on basic health literacy screening test(s). HL skills and perceived needs for support were identified through thematic analysis. Women appeared to be highly engaged in the decision-making process. They mentioned searching and selecting general information about pregnancy and labor, constructing their preferences based on their own pre-existing knowledge and experiences and by discussions with partners and significant others. However, women with low basic skills and primigravida perceived difficulties in finding reliable information, understanding probabilistic information, constructing preferences based on benefit/harm information and preparing for consultations. Women also emphasized dealing with uncertainties, changing circumstances of pregnancy and labor, and emotions. Maternity care professionals could further support clients by guiding them towards reliable information. To facilitate participation in decision-making, preparing women for consultations (e.g., agenda setting) and supporting them in a timely manner to understand benefit/harm information seem important.

## 1. Introduction

Clients (i.e., women and partners) in maternity care are expected to participate in decisions about available options during pregnancy and labor [1]. For many decisions in maternity care, e.g., on pain relief during labor, place and mode of labor, infant-feeding, or end of puerperium, women’s own values are important [2]. These decisions are often referred to as ‘preference-sensitive’ decisions, for which decision-making is not reliant on clinical expertise alone, but also on a woman’s own preferences related to perceived benefits and harms. In Western society, there is an ethical consensus that decisions during pregnancy are left primarily to the pregnant woman [3]. A large majority of women (95.2%) in maternity care indeed prefer to be the lead decision-maker [4].

A typical decision-making process requires women to be able to access, understand and apply benefit/harm information to make informed decisions, as well as to communicate effectively and exchange information with maternity care professionals, such as midwives, obstetricians and maternity care nurses [5]. These skills correspond to the broader concept of “health literacy” (HL) [6]. Health literacy can be divided into three categories: functional, communicative and interactive skills. Functional HL describes basic literacy skills to obtain relevant health information, and to apply that knowledge to a range of prescribed daily activities (e.g., reading medication labels) [7]. Interactive HL describes skills to obtain, understand and apply health information, and to interact with others to obtain information and make decisions. Critical HL describes skills to analyze information and to apply this information to gain control over life events and situations that impact one’s health [7]. McCaffery and colleagues specified essential HL skills of the decision-maker in five decision-making stages [8]. Translated to maternity care, this could mean the following. Stage 1 involves reading and interpreting information about pregnancy and labor procedures. This requires an understanding of associated medical terminology. Stage 2 requires women to understand different benefits and harms (including risks) of different options, such as options for pain relief during labor, options for infant feeding, or labor options after having had a caesarean in a previous pregnancy. Stage 2 also involves the interpretation of probability information, such as the prevalence of uterine scar rupture when attempting vaginal birth after caesarean section (0.72%) [9]. Stage 3 involves clients identifying their preferences for different benefits and harms and to combine this with provided probability information. To identify preferences, women may share and communicate values to significant others and anticipate future health states. Stage 4 necessitates women to participate in decision-making along with their care professional, to express their preference and articulate choice. For this purpose, women need to understand that choice and involvement in choice is possible, and they need to have the confidence to ask questions and negotiate with professionals. Implementing a decision (stage 5) requires social and psychological skills including self-efficacy, empowerment and motivation, as well as the removal of practical and financial barriers [8].

Maternity care professionals usually play an important role in helping women to find and interpret information of available options. They also typically support pregnant women to make their own decisions during pregnancy, birth and postnatal [1]. As client participation in decision-making has gained ground, professionals also increasingly provide their support through a shared decision-making (SDM) process. SDM offers opportunities for greater mutual understanding through a dialogue between client and care professional. The emphasis is on the process of coming to a decision [1].

Previous research showed that SDM positively influences satisfaction, adherence and well-being among patients making longer-term decisions and/or having chronic diseases [9]. SDM practiced in maternity care is also known to produce those beneficial effects, e.g., to reduce decisional conflict among clients, and to generate more positive feelings towards the new-born and fewer depressive symptoms [10]. Despite the progress made, adequate SDM implementation remains challenging in maternity care. Clients in general seem to perceive a low involvement in decision-making, e.g., as to decisions about labor induction, assisted vaginal birth and unplanned caesarean section [11]. A previous study on the SDM needs of clients and professionals in Dutch maternity care concluded that support was needed in order to improve communication skills of clients and professionals [12]. However, lower educated participants, who often have lower HL skills, were underrepresented in this study.

There has been little attention for SDM practiced in the maternity care setting and the specific needs and skills in decision-making of clients. Therefore, this qualitative study aimed to explore skills and needs for support regarding SDM in maternity care, by building on the perspective of clients. The research questions were:

1. What health literacy skills do women in maternity care use for (shared) decision-making?
2. What needs for support in shared decision-making do clients in maternity care perceive?

## 2. Materials and Methods

### 2.1. Study Design

In this qualitative study, we conducted individual face-to-face interviews with clients in maternity care from March to July 2019 in the Netherlands. A qualitative design was chosen to explore the perspectives of clients regarding skills they use in the decision-making process and their needs for support in SDM. According to Dutch law, this study was waived from requiring medical ethical approval (W18_307). We guaranteed the anonymity of the participants and ensured that written informed consent was obtained from all participants prior to conducting the interviews.

### 2.2. Research Population

The research population involved (pregnant) women with varying reading, understanding and calculating skills in a health context or ‘basic skills’ to achieve a representative sample of clients. 

Women with a gestational age of 25 weeks onwards and women who had given birth in the past four months were included. From 25 weeks of pregnancy, women would have had enough experiences to discuss decisions they have or had to make during their pregnancy, e.g., on pain relief during labor or place and mode of labor. To discuss the decisions that women make after birth (e.g., breast or bottle feeding), we chose to include women until four months after birth.

### 2.3. Data Collection

Participants were recruited from primary and secondary care practices in representative areas in the Netherlands for a mixed demographic using various strategies. Childbirth classes and pregnancy courses were visited by the first author (L.M.) to recruit pregnant women personally. Participants were also personally recruited in the waiting rooms of midwifery practices and child health clinics. Maternity nurses and obstetricians were asked to inform eligible women about our study and sent their contact details to the research team after receiving the consent of their client. The interviewer (L.M.) contacted the client for further information about the study and to make an appointment. We also used an online approach through a Dutch client organization of pregnant women and mothers (Geboorte Beweging) and social media.

### 2.4. Procedure and Measures

The qualitative interviews took on average 0.5–1 h each (time to fill in the questionnaire and to take the basic HL screening tests is excluded in the interview time) and took place at a location the participant preferred, such as her home. A translator was present during one interview. Clients’ partners were allowed to be present during interviews, but did not participate. We estimated women’s basic understanding and reading skills with the Short Assessment of Health Literacy-Dutch (SAHL-D) and calculating skills with the Newest Vital Sign-Dutch (NVS-D) (see below). We used two measures to assess self-reported HL of a non-Dutch speaking participant, because it was not possible to measure basic reading, understanding and calculating skills [13,14]. Basic skills (reading, understanding and calculating) were initially assessed after the interview as a background characteristic, but in the course of the recruitment process, initial convenience sampling shifted to purposive sampling to ensure that women with low basic skills were adequately represented and pregnant and postpartum women were equally distributed. Therefore, reading, understanding and calculating skills were later measured before the interview to select individuals with lower basic levels. Professionals were instructed to refer clients with low basic skills to the research team. Women who scored higher than the predefined cut-off points of SAHL-D and NVS-D were excluded.

#### 2.4.1. Interview Guide

**Figure A1:**
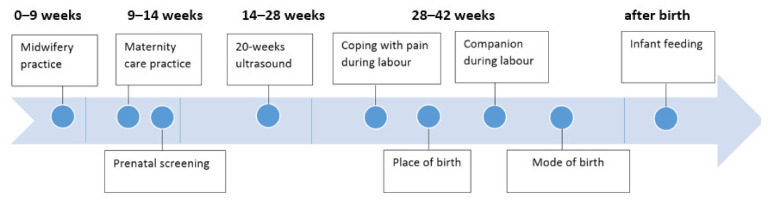
Pregnancy timeline presented during interviews.

McCaffery’s framework [8] was used as a conceptual framework in this study, because it combines the decision-making stages with required skills and competencies for each decision-making stage. The framework was used to construct our interview guide. The interview guide consisted of conversationally worded open questions and was iteratively adapted as the interviews progressed. The interview covered three broad topics of interest: women’s (1) perceived skills for decision-making, (2) experiences with SDM and (3) needs for support in SDM. To ensure a natural conversation, women were first asked what decisions they had to make or were expecting to make during pregnancy and after birth. We used a visual timeline to support this process (see Figure A1, Appendix A). The visual timeline presented several decisions clients need to make, ranging from prenatal screening, to decisions concerning the mode of birth. Next, we asked them what decision was most difficult for them to make. The decisions mentioned were used in the course of the interview to facilitate discussions. To start the discussion about SDM (topics 2 and 3), we briefly introduced a simple SDM model consisting of three essential steps: option, choice and decision talk [15]. Women were then asked whether they recognized the SDM steps in their decision-making process. Nearing the end of the interview, the participant was presented with examples of SDM tools to discuss her needs for support (topic 3). 

#### 2.4.2. Background Characteristics

The following variables were assessed orally before the interview: educational level, ethnic background and relationship status. Pregnancy-related outcomes assessed before the interview included: parity, gestational age or the number of weeks postpartum.

Measures that are often applied to assess performance-based basic literacy and numeracy skills are the SAHL-D and NVS-D. The SAHL-D assesses both word recognition and comprehension in the health domain. It contains single words that refer to medical specialties, tests, treatments and symptoms. Participants have to select the correct meaning of each word, using a multiple-choice format. For the SAHL-D, individuals with <9 out of 13 correct responses were considered as having low reading and understanding skills [16]. The NVS-D is a 6-question tool to assess an individual’s ability to find and interpret information (both text and numerical information) on an ice cream nutrition label. Individuals with <4 correct responses were considered to have low calculating skills [17,18].

We used two measures to assess self-reported HL of a non-Dutch speaking participant. The set of brief screening questions (SBSQ) in Dutch is a self-reported measure focusing on completing medical forms and reading and understanding medical information. An average score of ≤2 indicates inadequate HL [13]. The Perceived Efficacy in Patient-Physician Interactions questionnaire (PEPPI-5) is also a Dutch subjective measure, which assesses perceived sense of patients’ confidence when interacting with their physicians [14]. For the non-Dutch speaking participant, we assessed whether she scored below the predefined cut-off points of either SBSQ or PEPPI-5.

### 2.5. Data Analysis

All interviews were audio-recorded, transcribed literally by a professional organization and analyzed using thematic analysis [19]. The transcripts were coded in two stages: open coding with an inductive approach, and axial coding using the concepts of McCaffery’s framework for decision-making stages and HL skills [8]. This approach was chosen to ensure that we were not forcing our data within the predetermined constructs of the framework.

#### Coding and Analysis

All transcripts were coded by the first coder (L.M.). A second coder (M.E.D.) randomly and independently coded three transcripts in the first stage of coding. M.E.D. and L.M. agreed on the majority of the coding, and the remaining disagreements between authors were solved in consensus sessions. This was an iterative process. The new emergent codes were mapped into themes and subthemes. The decision-making stages (themes) and HL skills (subthemes) were categorized using the framework of decision-making stages and skills as a conceptual framework. Women’s needs for support were explored by analyzing how they used the identified skills and by exploring the difficulties they experienced when using these. Furthermore, we also assessed their perceived needs. We compared the emerging (sub)themes with the framework and assessed which HL skills were mentioned in the framework and not in the interviews, and vice versa. Next, themes and interpretations were discussed between researchers (M.P.F., E.M.A.S., O.C.D. and L.M.). The final coding scheme was applied to the entire dataset. The analysis was performed using MAXQDA 18.1.1. (VERBI Software, Berlin, Germany).

## 3. Results

### 3.1. Background Characteristics

In total, 51 women intended to participate and 22 were excluded because of no response or withdrawal. Six women scored high on SAHL-D and/or NVS-D and were excluded when convenience sampling shifted to purposive sampling. We included 23 clients, who were on average 33 years old. Ten participants scored low on either SAHL-D or NVS-D (Table 1). The participant who did not speak Dutch scored below the cut-off points of PEPPI-5 (average score 2) and SBSQ (average score 1). Eleven women were pregnant during the interview, of which five were primigravida and 12 women gave birth on average five weeks before the interview, of which five were primipara.

During the interviews, women mentioned the following decisions: which pregnancy courses to follow, diabetes test, induction of labor, maternal pertussis vaccination and options for breech birth (external cephalic version or planned caesarean section). In addition, the following decisions were discussed presented in the timeline: place of birth (hospital or at home, and which hospital) (discussed 14 times), prenatal screening for Down syndrome (first trimester combined test (FCT) or Non-Invasive Prenatal Testing (NIPT)) (discussed 11 times), pain relief during labor (discussed 11 times), infant feeding (bottle or breastfeeding) (discussed 9 times), healthcare providers (choice between midwifery and maternity care practices) (discussed 7 times) and twenty-weeks ultrasound (discussed 3 times). 

### 3.2. Stage 1: Understanding Pregnancy and the Procedures of Labor 

#### 3.2.1. Skills Stage 1

##### Find Sources of Information about Pregnancy and Labor

Women explained that they collected general information about pregnancy from various sources (see Table 2 for an overview of HL skills), such as online applications, their encounters with professionals, pregnancy courses, childbirth classes and encounters with other pregnant women (e.g., friends and relatives). Women with low basic skills tended to look for experiences from other pregnant women, in addition to factual information. Women with higher basic skills appeared to prefer more factual information, such as risk information about pain relief options received from professionals or online sources. As shown in quote 1 (Table 3), some women mentioned using a web-based application to track the physical changes of pregnancy.

Women often stated that they used multiple sources of information to be reassured, to affirm their decision, to develop an overview of options available and/or to prepare for consultations with their professional. The skill ‘using multiple sources’ was brought forward by low basic skilled women as well as by women with higher basic skills. However, women with lower basic skills stated that they tended to use multiple sources when they were in doubt about their own decision and asked other peers and professionals for reassurance.

##### Select and Appraise (Online) Information

Women reported that they had appraised information on websites of hospitals, the National Public Health Institute or websites that professionals recommended as trustworthy. They mentioned that an excessive amount of online information is available and that they had to select trustworthy information. Women with higher basic skills stressed that they avoided online fora and social media for information, because they might get worried by reading negative experiences of peers and they wanted to avoid invalid information. Several low basic skilled women stated that they use social media. They reported social media groups for pregnant women to be trustworthy, because it was information provided by women themselves, who had experienced pregnancy and labor.

Some women stated that they did not want to gather a lot of information and decided to stop looking for more information. Women with higher basic skills stressed that they wanted to avoid an overload of information, whereas other women with higher and lower reading, understanding and calculating skills indicated that they wanted to receive more information from their professional, for instance about pain relief options. Non-active information seekers were mostly multigravidas, who often mentioned they felt experienced to make a decision. 

##### Interpret Written or Spoken Pregnancy-Related Terminology

Women with low and higher basic skills both stated that they were often not able to understand medical terminology used by professionals or online. However, women with higher basic skills stated to be able to derive the meaning of medical and pregnancy-related terms from the context, and this was facilitated by repetition in the course of pregnancy. Low basic skilled women reported to be able to derive meaning from context, but also that medical terminology, for instance about the NIPT test, remained abstract for them (quote 2, Table 3). 

#### 3.2.2. Perceived Needs for Support in Stage 1

Women expected their professional to recommend websites with reliable information (quote 11, Table 4). They preferred information to be gathered from one source, which would make the information easily accessible. Women also expressed needs for clear and visual information in plain language, which they could read at home. One woman expressed a need for online decision aids that could help her decide which option would be suitable for her personal situation. Some women expressed that they received and read a lot of information about healthcare services and preferred a clear overview.

### 3.3. Stage 2: Understanding the Consequences—Risks, Limitations, Benefits and Uncertainties

#### 3.3.1. Skills Stage 2

##### Understanding Different Harms and Benefits of Options

Most women perceived themselves as being able to understand the harms and benefits of options which they had looked up themselves (mostly women with higher basic skills), had received from their maternity care professional or from peers (both women with low and higher basic skills). Low basic skilled women mentioned that the harms related to pain relief (2 clients), breech birth (1 client) and induction of labor (2 clients) were unclear, which made it difficult for them to decide. High basic skilled women did not mention difficulties in understanding harms and benefits, except for one woman who mentioned that the side effects of a maternal pertussis vaccination were unclear.

##### Understanding the Likelihood of Harms Occurring to Mother and/or Child

One woman with low basic skills and two women with higher basic skills seemed to estimate the likelihood of harms (e.g., contracting pertussis) occurring by using heuristics to estimate the risks, for example by estimating how many clients or children they knew who had experienced a certain event. Both high and low basic skilled women explained that they found it important to know probabilities about harms, but also stressed that they had experienced difficulties in interpreting them. One low basic skilled woman explained that risk information provided on the National Public Health Institute’s website was abstract and therefore without meaning for her (quote 3, Table 3).

##### Compare Options against Each Other

Both low and higher basic skilled women mentioned having compared options in two ways. Some looked up the advantages and disadvantages of all available options and discussed the information with their partner and/or professional, others compared the information they gathered from various sources themselves. 

#### 3.3.2. Perceived Needs for Support Stage 2

Information about benefits and harms was in general perceived as important by women with low and higher basic skills to be able to make decisions. Women expressed that they needed more benefit/harm information, for example about caesarean section when the baby is in a breech presentation. After seeing the decision aids during the interview, three women explicitly expressed understanding benefits and harms better when it is presented in an overview. Most women preferred to receive benefit/harm information from their professional rather than online, because they trust their health professional’s expertise. To understand the likelihood of harms occurring, some women preferred to read stories of other women, whereas others preferred factual information (quote 12, Table 4). 

### 3.4. Stage 3: Identifying Salient Preferences and Combining Utilities with Probabilities

#### 3.4.1. Skills Stage 3

##### Anticipating Health States during Labor or after Birth

Women, and especially those with higher basic skills, said to have taken potential future consequences for themselves and their child into account in their decisions. They talked about preparing themselves for future decisions beforehand, by imagining different scenarios and deciding what they would do when they would need to decide. For example, one woman explained that she would consider caesarean section if her child’s weight increased and a vaginal birth became more difficult.

##### Identifying Preferences for Different Outcomes

Women with low and higher basic skills stressed that they had constructed their preferences for options after weighing advantages and disadvantages of those options. Primigravidas found it especially difficult to identify their preferences, because they did not know what to expect from pregnancy and/or labor (quote 4, Table 3). Some women mentioned that they changed their preferences after obtaining information about the consequences of particular options, for example regarding the place of birth.

##### Combining Preferences with Probabilistic Information on Chance of Occurring

Only one woman with low basic skills and two women with higher basic skills explained having combined their preferences with probabilistic information in decision-making on mode of birth, pain relief and pertussis, for example on the chance of harms occurring, to affirm or change their decision (quote 5, Table 3).

##### Sharing and Communicating Values to Significant Others, Peers, Partner

Women mentioned discussing values regarding the importance of child’s and mother’s health, with their partner. They mentioned that their partner should be involved in the decision, because it involves their child’s health. However, some participants explained that women should make the decision themselves, because it is their body, and they know how it feels to give birth or breastfeed a child. Women did not seem to discuss their values with significant others and other pregnant women or women who gave birth recently, but almost all women mentioned sharing their experiences and options with them.

##### Using Previous Pregnancy and Birth Experiences and Own Knowledge

Women with low and higher basic skills both mentioned relying on previous experiences and knowledge in decision-making. They remembered information obtained from their first pregnancy, which they used in their current pregnancy. Multigravidas who had positive experiences from previous pregnancies and/or labor typically wanted to make the same decisions. Multigravidas who had negative experiences, in contrast, often said to reconsider their decisions in their current pregnancy. For example, one woman had a negative experience with her previous vaginal birth and wanted a caesarean section for her current birth (quote 6, Table 3).

#### 3.4.2. Perceived Support Needs Stage 3

To construct preferences about, for instance, breech birth or pain relief during labor, women mentioned the importance of receiving benefit/harm information in a timely manner (quote 13, Table 4). 

### 3.5. Stage 4: Participating in the Decision with Maternity Care Professional

#### 3.5.1. Skills Stage 4

##### Understanding that Involvement in Decision and Choice Is Possible

Generally, women appeared to be aware that they are involved in decision-making and wanted to be the lead decision-maker, some along with their partner. Mostly low basic skilled women mentioned that professionals presented choice and options. Only two women reported that they were not aware of the fact that they could choose, for example about breech labor, as shown in quote 7, Table 3. 

##### Articulate and Discuss Preference with Maternity Care Professional

Women mostly mentioned having had the opportunity to discuss preferences with their professional during regular check-ups, e.g., about pain relief, labor induction and mode of birth. Women did not mention discussing initial preferences with their, for example, midwife or obstetrician, but they did often report articulating their final choices to professionals. Most women said to have made several decisions beforehand (e.g., about place of birth), articulated their choice to their professional, and discussed their preferences in their conversation with the professional. 

##### Asking Questions to Maternity Care Professional

Women indicated that during their regular check-ups and postnatal care, they had asked questions about what they read to verify if that was correct. One participant, for example, explained that she had difficulties with breastfeeding and asked several care professionals for help (quote 8, Table 3). 

#### 3.5.2. Perceived Needs Stage 4

Not all women expressed that they were involved in the decision-making process. However, women with low and higher basic skills both expressed that, in hindsight, they found it important to be involved, and to create a sense of control. In addition, they emphasized this because they did not want to regret the decisions made (quote 14, Table 4). 

Women mentioned the importance of taking time to discuss advantages and disadvantages of options and their preferences with their partners. They wanted to prepare themselves better for regular check-ups and mentioned that agenda setting could help them to prepare for the consultation. Some women explicitly mentioned that professionals should respect and accept their decision about, for example, bottle feeding, and should not direct them towards another option. Women also expressed that during labor, a professional should make time to inform the client, guide them through labor and ask for consent for certain options (quote 15, Table 4). After seeing the decision aids in the interview, women explained that they needed such aids, especially to prompt questions and support decisions. 

### 3.6. Stage 5: Making a Decision 

#### 3.6.1. Skills

##### Self-Efficacy in Implementing a (Shared) Decision

Low basic skilled women mentioned more difficulties in making a decision compared to women with higher basic skills. Difficulties appeared to be mainly related to a perceived lack of information or to poor comprehension of information to make a decision. 

##### Cope with Practical Barriers of Options and Costs

Women mentioned practical and financial barriers related to choosing some of the options and mentioned that they assessed which options were suitable for their personal situation. Practical barriers concerned the unavailability of pain relief in hospitals, and the unavailability of various facilities to give birth (e.g., birth in bathtub). Most women were aware of the costs and the need for insurance related to certain options, such as receiving care of lactation consultants.

##### Taking Responsibility for Mother’s and Child’s Health

Women with low and higher basic skills both mentioned being motivated to be involved in decisions, because of the perceived importance of taking responsibility for their child. It was emphasized that this sense of responsibility made them motivated to gain control over their pregnancy and make well-informed decisions. Responsibility towards partners was also mentioned, i.e., making decisions not only for her child but also her partner’s child (quote 10, Table 3). Therefore, women wanted to consider all benefits and harms with the purpose of making the decision with the least harmful consequences. Women also mentioned that they were more accepting of certain risks to themselves, rather than for their child. 

#### 3.6.2. Perceived Needs Stage 5

One low basic skilled woman expressed the need to be empowered to make informed decisions during pregnancy (quote 16, Table 4). She wanted to be more assertive and ask more questions, in order to gain more information from her healthcare professional.

### 3.7. Other Decision-Making Skills

In addition to HL skills, we found the following skills, which could be relevant in more than one stage, in the decision-making process: coping with changes, uncertainty and emotions. 

#### 3.7.1. Coping with Changes

Women acknowledged that in certain situations, such as an unsafe situation for the child or in great pain, they might need to change their initial preferences. Situations typically mentioned were coping with the change when giving birth at home is not possible, that pain during labor becomes too much and when giving birth vaginally is not possible. Women mentioned to cope with those changes by accepting that changes in their preferences might occur, whereas other women mentioned that they would be disappointed if labor would not go as they expected.

#### 3.7.2. Coping with Uncertainty

Women expressed that they needed to cope with uncertainties related to the unpredictability of pregnancy and/or labor and the unpredictability related to harms occurring. Primiparous women also mentioned that they found it difficult to cope with their own feelings of uncertainty (quote 11, Table 3). Another example of uncertainties emphasized is not knowing whether they could receive the pain relief they preferred, because they were not certain in which hospital they would give birth. Women also did not know whether they would need pain relief, because they could not predict the pain. Some clients mentioned to be overall tolerant towards uncertainty. 

#### 3.7.3. Coping with Emotions

Four women mentioned that they had experienced difficulties to cope with their emotions during induction of labor, and changes in place and mode of birth. They felt overwhelmed and sometimes stressed by the amount of information they received from professionals in those situations. The emotions resulted in difficulties to make the accompanying decisions, for example, one participant mentioned the feeling of failure when she had to give consent for a caesarean section, when she wanted a vaginal birth. 

## 4. Discussion

This study aimed to explore health literacy (HL) skills and needs for support regarding participation in decision-making of clients in Dutch maternity care. Our findings showed that the HL skills discussed by women largely corresponded with the skills described in the framework of decision-making stages and HL skills developed by McCaffery et al. [8]. Additionally, several skills were specifically mentioned by women but not distinguished as such in the existing framework. These were: searching and selecting information, discussing initial preferences with health professionals as opposed to actual choices, since these are typically made with partners or postponed to the time of labor, and taking responsibility for mother’s and child’s health. In addition to HL skills, women also emphasized other decision-making skills, including dealing with changing circumstances during pregnancy and labor, uncertainties and emotions. Perceived needs included support in finding reliable information, in preparing for consultations (e.g., agenda setting) and in understanding and discussing benefit/harm information in a timely manner.

Women appeared to collect information about pregnancy and labor themselves from an early stage onward. Women with low and higher basic skills both searched for information online, however they appraised online sources in different ways. Women with higher basic skills explained to use websites of professional institutes (e.g., National Public Health Institute) and hospitals, whereas women with low basic skills seemed to look for experiences of peers (other pregnant women) online. This information might be easier to understand compared to information from professional institutes that often contains more medical jargon and statistical information. In our study, women with low basic skills explained that they considered anecdotal information, provided by women who went through pregnancy and labor, as reliable. However, the quality of these sources remains questionable, especially when it is used as the only information source. A previous systematic review also showed that low HL individuals use evaluation criteria that do not correspond to established quality criteria [20]. It is also known that lack of information can lead to searches in social media among first time mothers [21]. These findings together suggest that clients in maternity care need support or advice in selecting adequate information, as well as in interpreting the key messages communicated in information. 

McCaffery’s framework for decision-making stages and HL skills largely follows a rational decision-making model [8], based on the idea that people make decisions by making maximum use of information and weighing information elements according to one’s personal values and preferences. However, it is known that people often deviate from this rational decision-making model and use shortcuts and heuristics instead [22,23,24]. Similarly, our study showed that women construct their preferences using heuristics based on pre-existing knowledge gained from previous pregnancy and labor experiences. Unsurprisingly, primigravidas who cannot rely on previous birth experiences found it especially difficult to construct preferences, because they do not know what to expect from pregnancy or labor. This could pose problems in particular for primigravidas who do not understand probabilistic information, because they cannot rely on previous experiences either. Interviewed clients also explicitly expressed a need to better understand probability information related to the available options. They also seemed to look for information with affective meaning, e.g., experiences from peers. These findings imply that efforts should be made to provide women with well-balanced and easy to understand information, especially benefit/harm information. Probability information should be put into context, to provide women with information that has intuitive and affective meaning [25]. Narrative communications as sometimes used in decision aids [26] might provide such affective meaning. However, narrative content should be carefully constructed given its potential effect to bias decision-making when one-sided information is presented [27]. 

We found that women experienced difficulties coping with uncertainty and changing (medical) circumstances, which in turn led to emotional reactions. Women experienced uncertainty related to the unpredictability of pregnancy and/or labor as well as uncertainty related to the unpredictability related to harms occurring. Another study also showed that especially first-time mothers experience uncertainty, e.g., about physical and emotional reactions during labor and birth [28]. Maternity care professionals could play an important role in preparing women for such situations and re-adapting women’s preferences to the actual situation. In the present study, women mentioned to be disappointed when labor would not proceed as expected, whereas others were tolerant towards such changing circumstances. Therefore, pregnant women should be introduced to this narrative of changing circumstances in advance of labor, to remain flexible if the need arises [29]. The birth plan could be actively reconsidered and revised by women and professionals together, if they can no longer adhere to the original plan [28].

Primiparous women also dealt with their own uncertainties being in limbo, which was also shown by Soltani et al [30]. Their study suggested that the everyday experiences of first-time mothers were filled with challenges due to short-term tensions, fears and experienced limitations. As a result, participants experienced doubt about their ability to give birth, or to accept motherhood responsibilities [30]. Our participants mentioned a need for support by their professionals to prepare for decisions, for example by introducing the decisions that need to be made through agenda setting. Planning and preparation of women should be considered by professionals during pregnancy to reduce fears related to childbirth due to a lack of knowledge and inexperience [30]. 

### Strength and Limitations

A strength of our research is that we managed to represent the general population by including clients with various reading, understanding and calculating levels. However, the mean SAHL-D and NVS-D scores among our low basic skilled group (6 for SAHL-D and 2 for NVS-D) show that we were not able to include the group with lowest basic skills. The group with lowest basic skills might experience more difficulties in applying HL skills to make decisions and may need a different type of support than perceived by our study population.

## 5. Practice Implication

Maternity care professionals could further support women in participating in important decisions during pregnancy and labor. Guiding them towards reliable and easy to understand information is an important first step. Furthermore, preparing women explicitly for consultations (e.g., agenda setting), supporting them in understanding and discussing benefit/harm information seem important to facilitate participation in decision-making. To support women in coping with uncertainty and changing circumstances, a narrative can be adopted that emphasizes the ability to change preferences over time in advance of labor. For example, peers’ coping strategies according to changing circumstances could be presented in decision aids. However, narratives should be carefully designed, because it might also bias decision-making.

Future research should gain insight into strategies to provide affective meaning to probabilistic information, consequently, to support comprehension of women by meeting their current decision-making style. Our study did not include individuals with the lowest HL levels. Therefore, future research should provide insight into the perceived needs of this group, since they may need a different type of support in (shared) decision-making.

## 6. Conclusions

Women with various reading, understanding and calculating levels appeared to be highly engaged in the decision-making process. They mentioned to search and select general information about pregnancy and labor, and to construct their preferences based on their own pre-existing knowledge and experiences, and by discussions with partners and significant others. However, not all women were able to find reliable information, understand probabilistic information, construct preferences based on benefit/harm information and to cope with changing circumstances and uncertainties. Especially low basic skilled women and primigravidas perceived needs for support to apply these skills.

## Figures and Tables

**Table 1:** Background characteristics of interview participants (*n* = 23).

Background Characteristics	*n* (%)	Mean (SD *; Range)
Age		33 years (6; 20–45 years)
Educational level		
Low	4 (13%)	
Middle	2 (13%)	
High	17 (74%)	
Ethnic background		
Dutch	14 (61%)	
Non-Dutch: Western	2 (9%)	
Non-Dutch: Non-Western	7 (30%)	
Marital status		
Married/living together with partner	22 (96%)	
Basic skills		
SAHL-D *		8.8 (4; 0–13) **
NVS-D *		4.2 (2; 0–6) **
Low basic skills	10 (44%)	
Parity		
primigravida/primipara	10 (43.5%)	
multigravida/multipara	13 (56.5%)	
Number of weeks after birth		5 (2; 1,5–8)
Number of weeks pregnant		34 (4; 25–39)

** standard deviation (SD); Short Assessment of Health Literacy-Dutch (SAHL-D); Newest Vital Sign-Dutch (NVS-D).; * 1 missing.

**Table 2:** Decision-making stages and health literacy skills in maternity care.

Stage 1: Understanding Pregnancy Stages and the Procedures of Labor	Stage 2: Understanding the Consequences: Risks, Limitations, Benefits and Uncertainties	Stage 3: Identifying Preferences and Combining Utilities with Probabilities	Stage 4: Participate in Decision-Making with Maternity Care Professional	Stage 5: Make a Decision
(a) Find sources of information about pregnancy and labor	(a) Understand different harms and benefits of options	(a) Anticipate health states during labor or after birth	(a) Understand that involvement and choice is possible	(a) Self-efficacy
(b) Select and appraise (online) information -Decide when to stop looking for information	(b) Understand the likelihood of these occurring to mother and/or baby—carry out basic calculations	(b) Identify preferences for different outcomes	(b) Articulate and discuss preference to maternity care professional	(b) Taking responsibility for mother’s and child’s health
(c) Interpret written or spoken pregnancy-related terminology	(c) Interpret probabilities of harms occurring to mother and/or child	(c) Combine preferences with probabilistic information	(c) Ask questions to maternity care professional	(c) Cope with practical barriers of options and costs
	(d) Compare options against each other	(d) Share and communicate values to: -Significant others (e.g., friends, mother) -Peers -Partner		
		(e) Use own knowledge and previous pregnancy and birth experiences		

**Table 3:** Example quotes—decision-making stages and skills.

Stage 1: Understanding pregnancy stages and the procedures of labor	Quote 1—making sense of a condition: “... *the Pregnancy Plus app, and they also give you information from day to day, and week for week, about what you can approximately expect. And that gives you a lovely guideline for how your pregnancy is progressing.*” (high health literacy, 33 weeks pregnant, primigravida)
	Quote 2—interpreting spoken or written medical terminology: “*Yeah, it’s like you’re, I don’t know, trying to read the budget or something, really hard. Difficult language. I mean, I’m thinking, yeah but what am I really reading here?*” (low HL, 6 weeks postpartum, primiparous)
Stage 2: Understanding the consequences: risks, limitations, benefits and uncertainties	Quote 3—understanding the likelihood of harms occurring: “*Yeah, I think it’s too general. It really has to be simple, it has to be… you need to be able to put yourself in the place of someone that’s made that decision, why they did that. And not something like so-and-so percentage, no, that means nothing to me.*” (low HL, 6 weeks postpartum, primiparous)
Stage 3: Identifying preferences and combining utilities with probabilities	Quote 4—identifying preferences for different outcomes: “*That means thoroughly weighing up all sorts of things in advance, whilst of course I still haven’t got a clue how things are going to work out. So to me that’s very, very difficult*.” (high health literacy, 36 weeks pregnant, primigravida)
	Quote 5—combining preferences with probabilistic information: “*Well, at first I actually thought that I would prefer [to give birth] without pain relief, but when I read those risks, I thought, like, these are small things, and it is not extreme, but these are things that I think, like, if I don’t have to, then I don’t need to.*” (high HL, 39 weeks pregnant, primigravida)
	Quote 6—using knowledge and experiences: “*So besides finding it horrible the whole way things went during the childbirth, I found the aftermath horrible as well. And that made me decide... that I thought, like, that’s not something I want to deal with again.*” (high health literacy, 6 weeks postpartum, multiparous)
Stage 4: Participate in decision-making with maternity care professional	Quote 7—understanding that involvement and choice is possible: “*So do you want a caesarean or a breech birth? And that was completely new to us, new information. And that was a bit of a weird experience, because we didn’t even know we had any choice in the matter.*” (low HL, 38 weeks pregnant, primiparous)
	Quote 8—asking questions: “*…, but with breastfeeding, it did not go well immediately. I just sort of called in all backup, the midwife, the children’s healthcare left, the lactation consultant came over. Yeah, just take in all information that is available and that’s how I feel you decide.*” (high HL, 33 weeks pregnant, multiparous)
Stage 5: Make a decision	Quote 9—take responsibilities for mother and child’s health: “*In that case I’d indeed want to take more risks for myself than for the child, that’s for sure. Also, because it’s not just my child, it’s someone else’s child too. And I feel it deserves respect. Look, my own body, that’s my choice. But when it’s about somebody else’s child as well, then you’d never forgive yourself if something went wrong.*” (low HL, 38 weeks pregnant, primiparous)
	Quote 10—coping with uncertainty: “*Yeah, and that’s indeed awkward, because you’ve got no clue how things will work out and what it will be like. I mean, about position and mode of childbirth I can… I do have some ideas about that, but then I think, yeah, well, how will that really turn out… or will I still see it the same way then?*” (low HL, 38 weeks pregnant, primiparous)

**Table 4:** Example quotes—perceived needs for support.

Stage 1: Understanding pregnancy stages and the procedures of labor	Quote 11: “*Well, I really liked that the obstetrician gave us a website, because it was instantly clear, like okay, this information is apparently trustworthy, because she said so.*” (low HL, 38 weeks pregnant, primigravida)
Stage 2: Understanding the consequences: risks, limitations, benefits and uncertainties	Quote 12: “*Yes, I really like this, because I quite like to see facts, like 2 out of 1000 babies die around childbirth. That’s what I want to know: how many… not that there’s some chance, no, I want to know, okay, 2 out of 1000, as compared to (if we’re talking about caesarean) less than 0.5 of the 1000 babies die. So then you see it quite clearly, 0.2% or 0.05%, that’s what I want to know. How big is the difference, what are we talking about? So I think it’s great that this gives the real facts.*” (low HL, 38 weeks pregnant, primigravida)
Stage 3: Identifying preferences and combining utilities with probabilities	Quote 13: “*And there wasn’t a conversation about these are the cons or these are the pros, this would be good for you, or this would not be good…And it was at a moment that I could not think clearly anymore, because I did not sleep for three days and I was devastated in pain.*” (high HL, 2.5 weeks postpartum, primiparous)
Stage 4: Participate in decision-making with maternity care professional	Quote 14: “*Yes, I’m really pleased about that too, also because indeed it’s my own body and my baby, who of course might be absorbing medication too. So I’m really glad to have that freedom of choice and that I could make that decision.*” (high HL, 1.5 weeks postpartum, primiparous)
	Quote 15: “*The obstetrician who was there said after an hour, like, he hasn’t moved a single millimeter, and a first child is usually born after two hours of pushing. But I don’t really see that happening, that he’ll start moving in the second hour… So I’d advise you to just stop now, then I’ll just start the procedure for you now. And I was happy about that, I thought it was a good way of explaining. So then I thought, okay fine.*” (high HL, 2.5 weeks postpartum, primiparous)
Stage 5: Make a decision	Quote 16: “*So yeah, information can be difficult. Or like, as I was just saying, the information you get is, like, these are the pros and these are the cons and good luck. And then if you ask more questions later or you start discussing it further, then you do get some more information about it, and you do understand it better. So then I think that… yeah, that perhaps we could have been more assertive ourselves about that, and make an additional appointment to discuss it further. I’m not sure about that, but suddenly everything had to go so fast.*” (low HL, 38 weeks pregnant, primiparous)

## Data Availability

The data presented in this study are available on request from the corresponding author, provided that approval from the research group is obtained and agreement is made on data security, data transfer, period of data availability, and possible use of data (for commercial ends), costs for use of data and methodology. The data are not publicly available due to privacy of participants.
